# Adherence to Mediterranean and low-fat diets among heart and lung transplant recipients: a randomized feasibility study

**DOI:** 10.1186/s12937-018-0337-y

**Published:** 2018-02-14

**Authors:** Timothy R. Entwistle, Adèle C. Green, James E. Fildes, Kyoko Miura

**Affiliations:** 1 The Transplant Centre, University Hospital of South Manchester, Manchester, M23 9LT UK; 2 Cancer and Population Studies Group, QIMR Berghofer Medical Research Institute, 300 Herston Road, Herston, QLD 4006 Australia; 3 CRUK Manchester Institute, University of Manchester, Wilmslow Road, Manchester, UK; 4 Manchester Collaborative Centre for Inflammation Research, University of Manchester, Manchester, UK

**Keywords:** Mediterranean diet, Low fat diet, Dietary compliance, Randomized study, Pilot projects, Organ transplant recipients

## Abstract

**Background:**

Heart and lung transplant recipients are at a substantially increased risk of cardiovascular disease (CVD). Since both low-fat and Mediterranean diets can reduce CVD in immunocompetent people at high risk, we assessed adherence among thoracic transplant recipients allocated to one or other of these diets for 12 months.

**Methods:**

Forty-one transplant recipients (20 heart; 21 lung) randomized to a Mediterranean or a low-fat diet for 12 months received diet-specific education at baseline. Adherence was primarily assessed by questionnaire: 14-point Mediterranean diet (score 0–14) and 9-point low-fat diet (score 0–16) respectively, high scores indicating greater adherence. Median scores at baseline, 6 months, 12 months, and 6-weeks post-intervention were compared by dietary group. We further assessed changes in weight, body mass index (BMI) and serum triglycerides from baseline to 12 months as an additional indicator of adherence.

**Results:**

In those randomized to a Mediterranean diet, median scores increased from 4 (range 1–9) at baseline, to 10 (range 6–14) at 6-months and were maintained at 12 months, and also at 6-weeks post-intervention (median 10, range 6–14). Body weight, BMI and serum triglycerides decreased over the 12-month intervention period (mean weight − 1.8 kg, BMI –0.5 kg/m^2^, triglycerides − 0.17 mmol/L). In the low-fat diet group, median scores were 11 (range 9–14) at baseline; slightly increased to 12 (range 9–16) at 6 months, and maintained at 12 months and 6 weeks post-intervention (median 12, range 8–15). Mean changes in weight, BMI and triglycerides were − 0.2 kg, 0.0 kg/m^2^ and − 0.44 mmol/L, respectively.

**Conclusions:**

Thoracic transplant recipients adhered to Mediterranean and low-fat dietary interventions. The change from baseline eating habits was notable at 6 months; and this change was maintained at 12 months and 6 weeks post-intervention in both Mediterranean diet and low-fat diet groups. Dietary interventions based on comprehensive, well-supported education sessions targeted to both patients and their family members are crucial to success. Such nutritional strategies can help in the management of their substantial CVD risk.

**Trial registration:**

The IRAS trial registry (ISRCTN63500150). Date of registration 27 July 2016. Retrospectively registered.

**Electronic supplementary material:**

The online version of this article (10.1186/s12937-018-0337-y) contains supplementary material, which is available to authorized users.

## Background

Cardio-metabolic disturbance is common in heart and lung transplant recipients and is associated with cardiovascular disease (CVD)-related morbidity and mortality [1, 2]. Despite careful patient management, blood pressure and blood lipids tend to rise after transplantation such that 5 years after heart transplantation, the cumulative incidence rates of hypertension and hyperlipidemia are 92 and 88% respectively [1], and similar after lung transplantation [2]. Overweight/obesity are known predictors for these conditions, and excessive weight gain occurs frequently post-transplantation [3, 4]. Several studies have shown dramatic upward weight trajectories in organ transplant recipients in the post-transplant period [5] with, for example, an average 10 kg weight gain in the first year in heart transplant recipients [3]. Factors contributing to this weight gain include altered energy metabolism [6] and side-effects of medications [7]. To prevent obesity and reduce the risk of associated chronic conditions in the general population, dietary modification is fundamental. Two dietary regimens have been shown to reduce CVD risk: the low-fat diet and the Mediterranean diet [8].

In contrast, in immunosuppressed populations, current CVD management focuses on tailoring immunosuppression and drug treatment [9]; only a limited number of studies have shown dietary approaches to be effective in organ transplant populations [10–12]. As a consequence, little is known about adherence to dietary interventions following transplantation [12], although it is recognized that in general, non-adherence to interventions is common and limits their overall effectiveness [13]. We therefore performed this study to assess adherence in thoracic transplant recipients randomly assigned to either of the two dietary interventions known to reduce CVD risk factors. We also assessed whether transplant recipients maintained these dietary changes after cessation of the intervention.

## Methods

The Assessment of the MEditerraneaN Diet In heart and lung Transplantation (AMEND-IT) study was a single-center parallel-randomized study designed to assess the feasibility and acceptability of two dietary interventions, namely the Mediterranean diet and low-fat diet among heart and lung transplant recipients. The 12-month study was conducted at the University Hospital of South Manchester. Eligible participants were clinically stable, aged ≥16 years, and a minimum 6 months post-transplant. Exclusion criteria included acute rejection, infection, prevalent cancer, diabetes, or chronic kidney disease (estimated glomerular filtration rate ≤ 30). Patients with any competing dietary issues (i.e. food allergies and following medically prescribed diets that conflicted with the interventions) were also excluded.

**Fig. 1:**
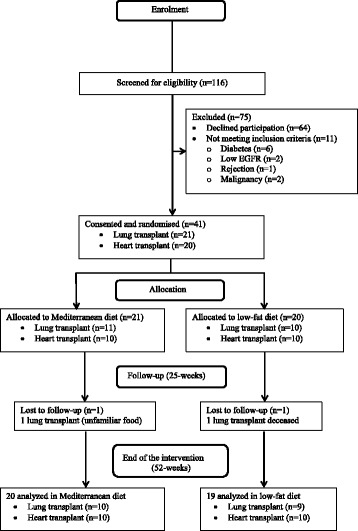
CONSORT flow diagram for AMEND-IT study. EGFR: estimated glomerular filtration rate

Study participants were identified through hospital records at the transplant outpatient clinic and recruitment commenced in February 2014 and ended in October 2014. A total of 116 heart or lung transplant recipients meeting the criteria were contacted. Each received an information package that included a participant information sheet, contact details and a return form. Among those contacted, 75 patients were not included (64 declined participation, 11 did not meet inclusion criteria) and the remaining 41 (20 heart, 21 lung) gave written consent to participate (Fig. 1). The study was approved by the NRES Committee North West (REC reference number 13/NW/0310) and was retrospectively registered on the IRAS trial registry (ISRCTN63500150).

Participants were stratified according to organ type and transplant date, and then randomly assigned to either a Mediterranean diet or a low-fat diet intervention using a computerized system with random block size and an equal 1:1 allocation ratio. To blind the investigator during recruitment, randomized codes were sent to a third person who then allocated the randomized interventions to patients per protocol.

### Dietary intervention

The study sought to change patients’ overall dietary habits through behavioral modification. Several 5-h group education sessions were conducted for each diet group (with an accompanying family member if desired) on specified dates outside routine outpatient visits. A nutritionally-trained investigator administered the education sessions and explained the scientific rationale in a visual, interactive manner, and advice given about preparation and storage of fresh, whole foods as relevant. A trained chef demonstrated practical methods for Mediterranean or low-fat meal preparation. Attendance at each session was restricted to maximum of 10–12 participants (excluding family members). Energy intake restriction was not explicit, but portion sizes were discussed throughout the study. Each participant was encouraged to attend this baseline education session with an adult member of the same household [14]. All participants received a printed booklet containing advice about shopping, food preparation, hygiene, storage, dining out and recipes. Additional advice and support were provided at 6- and 12-month outpatient visits, and during six 15-min telephone consultations spaced evenly through the intervention period, when participants could raise any questions or concerns and when key dietary recommendations (e.g. plant-based diet, consume minimally processed food) were reinforced. SMS messaging was also used to remind patients of clinic study requirements.

Participants allocated to the Mediterranean diet received information and encouragement to follow an eating pattern representative of a traditional Mediterranean diet [15]. The key dietary recommendations were: daily mixed consumption of a range of vegetables, fruit, wholegrains, fish/seafood, raw nuts and legumes; abundant use of extra-virgin olive oil (a free 5-l container of extra-virgin olive oil was provided to each participant); moderate consumption of dairy products and red wine; low intake of red and processed meats, of sweets, sweet-baked pastries and sweetened beverages.

Participants assigned to the low-fat diet were advised to follow modified British Heart Foundation low-fat guidelines [8] with an emphasis on consuming mainly plant-based wholefoods similar to the Mediterranean diet,with advice to minimize high-fat foods such as processed meats, commercially baked pastries and desserts, and vegetable oils and spreads. Advice was given on how to identify and avoid different types of fat. Each participant received a low-fat recipe book. The main difference between the two diets was the intake of oil and fat which was encouraged to a moderate degree in the Mediterranean diet but discouraged in the low-fat diet.

### Dietary assessment

Participants were asked to complete intervention-specific short dietary questionnaires at baseline, 6- and 12-months and again 6-weeks after the intervention to determine short-term post-study adherence. These questionnaires were completed at the hospital during routine visits, except for the post-intervention questionnaire that was sent by mail and completed at home.

Adherence to the Mediterranean diet was measured using a 14-point Mediterranean diet-screening questionnaire, adapted from the previously developed and validated version used in the Prevención con Dieta Mediterránea (PREDIMED) study conducted in a high CVD risk population [15]. The short Mediterranean diet questionnaire contains 14 questions characterizing key food groups commonly consumed in a traditional Mediterranean diet (Additional file 1) [15]. Favorable responses (‘yes’) were assigned a value of ‘1’; ‘no’ was assigned ‘0’ and answers were summed to a total Mediterranean diet score ranging from 0 to 14, with higher scores indicating greater adherence. A validation study among a separate sample of 16 heart and lung transplant outpatients demonstrated good agreement with the Mediterranean diet score derived from the 183-item previously validated self-administered semi-quantitative food frequency questionnaire (FFQ) [16]. The mean agreement expressed as a ratio (short questionnaire: FFQ) was 0.99 (95% limits of agreement 0.60–1.38) (ratio of 1.00 indicating perfect agreement) and the two one-sided t-test showed the scores derived from the two methods were equivalent.

For participants assigned to the low-fat diet, the 9-point short questionnaire also adapted from the PREDIMED study [15] was used to measure adherence. The 9-point low-fat diet questionnaire assessed consumption frequencies or serving size of seven food items and two items assessed dietary habits (Additional file 2). For questions that assessed food intake, there were three possible answers scoring ‘0–2’; with favorable responses receiving higher scores. For the two questions that assessed dietary habits, favorable responses (‘yes’) were assigned ‘1’and ‘no’ was assigned ‘0′. Resultant low-fat diet scores ranged from 0 to 16 with a higher score indicating greater adherence. The same validation study that assessed validity of the Mediterranean diet questionnaire also showed good agreement between the low-fat short questionnaire with the FFQ: mean agreement 1.04, 95% limits of agreement 0.12–0.79; and the two one-sided t-test showed results from the two methods were equivalent [16].

### Adherence index

A diet adherence index was created for each diet using the scores from the short adherence questionnaire rescaled to range from 0 to 100, to reflect percentage of score achieved. For example, low-fat diet participants scoring 16 received an adherence index of 100 (16/16 × 100), whereas those who scored 9 in the Mediterranean diet achieved an adherence index of 64 (9/14 × 100). This enabled comparisons of the patterns of adherence between the two dietary regimens.

### Anthropometric and laboratory measurements

We measured body weight and serum triglycerides at baseline and at the end of the intervention to provide objective measures of adherence to the allocated diets. Weight was measured wearing light clothing with calibrated scales. Body mass index (BMI) was calculated (kg/m^2^). Fasting blood samples were collected and processed immediately and stored at − 80 °C for later analysis. Triglycerides were quantified on an Architect c16000 immunoassay analyzer.

As a potential indicator of clinical effectiveness of the interventions, a biomarker of inflammatory state, namely high sensitive C-reactive protein (hs-CRP), was measured. However, hs-CRP values were highly skewed because inflammation status was heavily influenced by other factors, especially the background morbidity (generally high inflammation) and routine medication (Prednisolone, lowering inflammation) in these patients. Consequently, these data did not provide useful information by diet group.

### Statistical analysis

As this was a feasibility study, no formal sample size calculation was carried out. However, we aimed to enroll 40 to 50 participants, a number sufficient to indicate if the interventions were acceptable and the clinical evaluations feasible.

Statistical analyses were performed with SAS, version 9.4 (SAS Institute Inc. Cary, NC). Descriptive statistics were carried out and median (interquartile range (IQR)) was used for the continuous variables, and number (%) for ordinal or categorical variables. The Wilcoxon signed rank sum test was used to compare differences in the median baseline Mediterranean diet or low-fat diet scores, and the scores at each follow-up time. Taking into account the potential imbalanced baseline indices within the groups, ANCOVA was also used to assess adherence indices at each follow-up time compared with baseline indices [17]. Since this is a feasibility study, significance tests of differences between the two diet groups were not performed [18].

## Results

Among the 41 participants, one lung transplant recipient assigned to the Mediterranean diet was lost to follow-up due to dislike of unfamiliar food types and one lung transplant recipient in the low-fat diet group died from chronic rejection. As a result, *n* = 20 in the Mediterranean diet group (10 heart, 10 lung) and *n* = 19 in the low-fat diet group (10 heart, 9 lung) completed the study. In the Mediterranean diet group, 13 (65%) had a family member attend the education session and in the low-fat diet group 16 (84%) were accompanied by a family member. At baseline, the mean age of those randomized to the Mediterranean diet and low-fat diet groups was 56 and 54 years, respectively (Table 1). While body weight was slightly higher in the Mediterranean diet group, BMI was no different (29 kg/m^2^ for both groups). Similarly, the two groups had comparable waist circumference, systolic and diastolic blood pressure, and heart rates. All participants were on immunosuppressive medication and most were prescribed antihypertensive and/or cholesterol-lowering medications.

**Table 1:** Baseline characteristics of Mediterranean and low-fat diet groups (*N* = 41)

	Mediterranean (*n* = 21)	Low-fat (*n* = 20)
Age (year)[median (range)]	58 (33–65)	59 (27–65)
Male [n (%)]	15 (71)	14 (70)
Weight (kg ± SD)	87 ± 15	82 ± 16
BMI (kg/m^2^ ± SD)	29 ± 4	29 ± 5
Waist circumference (cm ± SD)	102 ± 12	100 ± 13
Systolic BP (mm Hg ± SD)	138 ± 13	141 ± 14
Diastolic BP (mm Hg ± SD)	86 ± 11	88 ± 8
Heart rate (bpm ± SD)	80 ± 13	79 ± 11
Immunosuppressive medication [n (%)]		
Cyclosporine	14 (67)	17 (85)
Tacrolimus	7 (33)	3 (15)
Everolimus	1 (5)	0 (0)
Mycophenolate	13 (62)	12 (60)
Azathioprine	5 (24)	5 (25)
Prednisolone	21 (100)	20 (100)
Other medication [n (%)]		
Antihypertensive agents	17 (81)	15 (75)
Cholesterol lowering medication	17 (81)	16 (80)
Organ transplantation [n (%)]		
Heart	10 (48)	10 (50)
Lung	11 (52)	10 (50)

At baseline, the median Mediterranean diet score was 4 (IQR 2) overall (Table 2). The score significantly increased to 10 at 6 months (IQR 3; *p* < 0.001) and remained elevated at 12 months and at 6 weeks post-intervention. For the low-fat diet group, the median baseline score was 11 (IQR 5); and increased to 12 (IQR 2; *p* < 0.001) at 6 months. The score remained high during and after the intervention.

**Table 2:** Median scores (interquartile range) from short dietary questionniare^1^ at each time point and the score differences from the baseline to each time point^2^

	Mediterranean diet (*n* = 20)		Low fat diet (*n* = 19)	
	Scores at each point	Differences from baseline	Scores at each point	Differences from baseline
Baseline				
All	4 (2)	–	11 (5)	–
Heart	4 (1)	–	11 (5)	–
Lung	4 (4)	–	10 (5)	–
6 months				
All	10 (3)	5 (3)***	12 (2)	2 (3)***
Heart	10 (3)	5 (3)**	13 (2)	3 (6)*
Lung	10 (3)	5 (2)**	12 (2)	2 (2)*
12 months				
All	9 (4)	4 (2)***	13 (3)	2 (4)**
Heart	11 (4)	5 (3)**	13 (3)	3 (3)*
Lung	9 (2)	4 (4)**	13 (3)	2 (2)*
6 weeks post-intervention				
All	10 (3)	5 (3)***	12 (2)	2 (4)*
Heart	10 (3)	5 (4)**	13 (2)	3 (5)*
Lung	10 (3)	5 (2)**	11 (2)	1 (3)

* *p* < 0.05; ** *p* < 0.01; *** *p* < 0.001

^1^Mediterranean diet score ranged from 0 to 14; low-fat diet score ranged from 0 to 16; higher scores indicate greater adherence

^2^*p*-values from Wilcoxon signed rank sum test

**Fig. 2:**
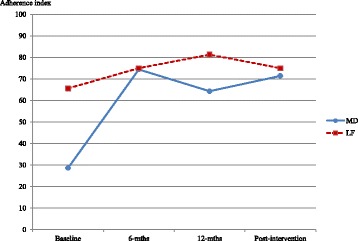
Median adherence indices by diet type over 58-weeks. MD: Mediterranean diet; LF: low-fat diet. Mediterranean diet *n* = 20 (10 heart, 10 lung); low-fat diet *n* = 19 (10 heart, 9 lung). Adherence index ranged from 0 to 100, reflecting % of score achieved. Mediterranean diet adherence index = (actual score observed/14) × 100; low-fat diet adherence index = (actual score observed/16) × 100. ANCOVA was used to assess adherence indices at each follow-up time point compared with baseline

The median adherence index at baseline was lower for the Mediterranean diet group (median 29) compared with the low-fat diet group (median 66) (Fig. 2). However, the Mediterranean diet adherence index increased to a level comparable to the low-fat diet group at each follow-up time point; and was maintained 6-weeks after the intervention ceased. A significant increase in indices at each time point from baseline were observed for both diet groups (all *p* < 0.001) and there were no statistically significant differences in transplant organ types in either intervention (all *p* > 0.05).

Adherence was objectively assessed by changes in body weight, BMI and serum triglycerides in the 12 months from baseline to the end of the intervention period. Compared with baseline body weight, there was a mean weight loss of 1.8 kg in the Mediterranean diet group (− 1.8 kg; 95% CI –4.6, 1.1) at 12 months, and negligible weight loss in the low-fat diet group (mean − 0.2 kg; 95% CI –2.4, 2.1). Similarly, BMI decreased in Mediterranean diet group from 29.0 to 28.5 kg/m^2^ (mean change − 0.5 kg/m^2^, 95% CI –1.4, 0.4) whereas no change was observed in the low-fat group over the 12 months (28.6 kg/m^2^ at both time point, mean change 0.0 kg/m^2^, 95% CI –0.8, 0.7). Over the same period, the serum triglycerides levels declined in both groups: Mediterranean diet − 0.17 mmol/L (mean − 9%, 95% CI –20, 4); low-fat diet − 0.44 mmol/L (mean − 21%, 95% CI –33 to − 7).

## Discussion

In this feasibility study comparing two dietary interventions in a thoracic transplant outpatient setting, both groups reported changes in their normal eating pattern and adhered to their allocated dietary regimen. In both Mediterranean diet and low-fat diet groups, the change from baseline eating habits was evident at 6 and 12 months; and this change was maintained 6 weeks after intervention.

The current evidence regarding dietary intervention and adherence among solid organ transplant recipients is limited [12]. Indeed this is the first known randomized study reporting adherence to different dietary interventions in heart or lung transplant recipients. One previous non-randomized study was conducted in 42 heart transplant recipients who were encouraged to follow the American Heart Foundation Step 1 Diet and adherence was assessed after 3 months, with only 50% adhering to the diet in that short-term study [10].

Non-adherence to dietary regimens in intervention studies is common and clearly hinders effectiveness [13]. Type of diet prescribed may influence adherence as a low-fat diet appears more difficult to follow and maintain compared with a moderate-fat diet [19]. However, in our study adherence did not differ between the groups and this may be partly due to the detailed advice given to the low-fat diet group. Overall fat and oil intake reduction was emphasized and practical advice was given about how to achieve this (e.g. shopping and cooking). Thus, the actual dietary advice, its delivery methods, and whether close patient support is on hand, appears very important. As highlighted by Zeltzer et al. [12], nutritional support following transplantation is currently sub-optimal and dietary advice is often too general and too-often provided without visual or practical information. To increase adherence, we used several methods: ensuring family support, practical and visual cooking advice, and educational sessions designed to emphasize the reasons why specific foods are beneficial whilst others contribute to disease progression. Our integrated and highly supportive approach may also explain the very low attrition observed during the 12-month intervention.

The PREDIMED study showed a 2-point increase in the Mediterranean diet score in a non-transplant population which was associated with a 14% reduction of all-cause mortality [20]. Similarly, a one-point increase in the Mediterranean diet adherence score was associated with an 18% reduction of myocardial infarction amongst a high risk CVD Mediterranean population [21]. Although there are differences in how diet adherence was assessed in these previous studies, our finding of a 5-point improvement is potentially clinically important among heart and lung transplant recipients.

The previous dietary intervention study of 42 heart transplant recipients encouraged consumption of a low-fat diet for 12 months [10]. This coincided with a reported beneficial effects on lipid and glucose regulation, weight loss and statin use in those who adhered, compared with non-adherent patients at 12 months and at 48-months follow-up [10]. These findings highlight the importance of adherence to diet regimens to help optimize health status. Further, in the present study we found baseline adherence index was much lower for the Mediterranean diet than the low-fat diet, likely reflecting the unfamiliarity of the Mediterranean diet in the UK as a non-Mediterranean European population [22] and the standard low-fat dietary advice previously given to study participants [23].

Adherence measured by body weight and serum triglycerides was further evidence of participants’ dietary changes. While the weight reduction observed appears small, without any intervention, post-operative organ transplant recipients weight changes are typically relentlessly upward [5]. Similarly, a rising trajectory of blood lipids including triglycerides is well documented among organ transplant recipients [1, 2]. Nonetheless, the level of triglycerides decreased in both diet groups indicating participants had followed their allocated diets. In particular, the findings of lowered serum triglycerides suggest our participants reduced energy intakes that were excessive.

Limitations included the assessment of adherence using short diet questionnaires. Although short questionnaires have been widely used and reflect adherence of specific diets in non-transplant population [24]; method has not yet been validated with biomarkers of dietary intakes among transplant population. The repeatability has also not been assessed. However, the relative validity of the diet short questionnaires was assessed against a FFQ and showed good agreement [16]. In addition, adherence was assessed using body weight and serum triglycerides as objectively measured clinical and biomarker outcomes: these indicated adherence had been maintained. Finally, although the results from this feasibility study may not be widely generalizable because of small sample size and thus likely not representative, the methods and findings should assist in planning similar intervention studies using short index-based adherence questionnaires.

## Conclusion

Based on our findings, implementation of Mediterranean diet or low-fat diet interventions among clinically stable heart or lung transplant recipients can be achieved, adhered to, and maintained throughout a 12-month period, and even in the short-term, post-intervention. Dietary interventions based on education sessions targeting both patients and family members are crucial for the interventions’ success. The educational approach with visual aids and practical information, along with the comprehensive support strategy, are likely to have assisted in patients’ adoption and maintenance of their allocated diets during and after the intervention. Findings from this study provide new evidence to inform nutritional support strategies in thoracic transplant recipients.

## Additional files

Additional file 1: **Table S1.** AMEND-IT 14-point Mediterranean dietary adherence questionnaire. (PDF 80 kb)

Additional file 2: **Table S2.** AMEND-IT 9-point low-fat diet adherence questionnaire. (PDF 79 kb)

## Abbreviations

AMEND-IT study: Assessment of the MEditerraneaN Diet In heart and lung Transplantation study
BMI: Body mass index
CI: Confidence intervals
FFQ: Food frequency questionnaire
hs-CRP: High sensitive C-reactive protein
IQR: Interquartile range
PREDIMED study: Prevención con Dieta Mediterránea study
