# Supplementary material for: Adherence to Mediterranean and low-fat diets among heart and lung transplant recipients: a randomized feasibility study
Source: Nutr J. 2018 Feb 14;17:22. doi: 10.1186/s12937-018-0337-y (PMC5813407; doi:10.1186/s12937-018-0337-y)
Supplement: Supplementary file 1 — Table S1. AMEND-IT 14-point Mediterranean dietary adherence questionnaire. (PDF 80 kb) [file 12937_2018_337_MOESM1_ESM.pdf]

Supplemental Table 1: AMEND-IT 14-point Mediterranean dietary adherence questionnaire[1]

Dietary habit/ consumption frequency	Response for 1 point[2]
1. Do you use extra virgin olive oil as main culinary/cooking fat/oil?	Yes
2. How much extra virgin olive oil do you eat in a given day (including oil used for cooking, salads, out-of-home meals etc.)?	≥ 4 table spoons
3. How many vegetable servings do you eat per day? (1 serving = 200 g; side dish as a half serving)	2 or more (at least 1 serving raw or as salad)
4. How many fruit units (including natural fruit juices) do you eat per day?	≥ 3
5. How many servings of red meat, beef-burger or meat products (ham, sausage, bacon etc.) do you eat per week?	< 1
6. How many servings of butter, margarine or cream do you eat per day? (1 serving = 12 g or 1 tablespoon)	< 1
7. How many sweetened and/or carbonated beverages do you drink per day?	< 1
8. How much red wine do you drink per week?	≥ 7 glasses
9. How many servings of legumes/pulses do you eat per week? (1 serving = 150 g or ½ cup in dry form)	≥ 3
10. How many servings of fish or shellfish do you eat per week? (1 serving 100–150 g of fish [size of deck of cards] or 4–5 units or 200 g of shellfish)	≥ 3
11. How many times per week do you eat desserts or pastries (not homemade), such as biscuits, cookies, cakes, custard etc.?	< 3
12. How many servings of unsalted nuts (including peanuts) do you eat per week? (1 serving = 30 g or 1 small handful)	≥ 3
13. Do you prefer to eat chicken, turkey or lean game instead of pork, beef-burger, sausage or bacon?	Yes
14. How many times per week do you eat a self-made sauce (sofrito) prepared with tomato, garlic, onion or leeks - simmered in olive oil. Used as a garnish to season vegetable, pasta or rice dishes?	≥ 2

[1] Adapted from Estruch et al.

[2] Criteria to score=1; otherwise score=0.

Reference

1. Estruch R, Ros E, Salas-Salvadó J, Covas M-I, Corella D, Arós F, Gómez-Gracia E, Ruiz-Gutiérrez V, Fiol M, Lapetra J, et al: Primary Prevention of Cardiovascular Disease with a Mediterranean Diet. N Engl J Med 2013, 368:1279-1290.
