# Supplementary material for: Adherence to Mediterranean and low-fat diets among heart and lung transplant recipients: a randomized feasibility study
Source: Nutr J. 2018 Feb 14;17:22. doi: 10.1186/s12937-018-0337-y (PMC5813407; doi:10.1186/s12937-018-0337-y)
Supplement: Supplementary file 2 — Table S2. AMEND-IT 9-point low-fat diet adherence questionnaire. (PDF 79 kb) [file 12937_2018_337_MOESM2_ESM.pdf]

Supplemental Table 2: AMEND-IT 9-point low-fat diet adherence questionnaire[1]

Dietary habit/ consumption frequency	Score		
	2	1	0
1. On average, how many tablespoons of vegetable oil (e.g. sunflower, olive, sesame, rapeseed oil) do you consume in a given day for frying, salads & out of house meals etc.?	0–1	2–4	≥5
2. Do you remove visible fat (or skin) from chicken, duck, pork, lamb or beef before cooking; and the fat from soups, broths and cooked meat dishes before eating?		Yes	No
3. How many servings of non-lean meats, hamburger, commercial minced meat, sausage, cold meat, cured ham, bacon, salami or offal do you consume per week? (meat serving = 100g or salami or bacon 30g)	0–1	2–4	≥5
4. How many servings of butter, margarine, lard, mayonnaise, milk-cream or milk-based ice cream do you consume per week? (spread fat serving= 2g, ice cream= 100g)	0–1	2–4	≥5
5. Do you exclusively consume low-fat dairy products?		Yes	No
6. How many times per week do you eat fried foods e.g. chips/French fries or fried chicken?	0–1	2–4	≥5
7. How many times per week do you consume fatty fish or fish or seafood canned in oil?	0–1	2–4	≥5
8. How many servings of commercial sweets or industrial bakery products (not homemade), such as cakes, cookies, biscuits or custard do you consume per week? (cake serving = 80g or 6 biscuits 40g)	0–1	2–4	≥5
9. How many times per week do you consume nuts (including peanuts), potato, corn or wheat crisps or commercial snacks?	0–1	2–4	≥5

[1] Adapted from Estruch et al.

Reference

1. Estruch R, Ros E, Salas-Salvadó J, Covas M-I, Corella D, Arós F, Gómez-Gracia E, Ruiz-Gutiérrez V, Fiol M, Lapetra J, et al: Primary Prevention of Cardiovascular Disease with a Mediterranean Diet. N Engl J Med 2013, 368:1279-1290.
